# Rare paratesticular aggressive angiomyxoma mimicking an epididymal tumor in an 82-year-old man: Case report

**DOI:** 10.1515/med-2021-0317

**Published:** 2021-06-30

**Authors:** Chi-Fang Chen, Tao-Yeuan Wang, Marcelo Chen, Yung-Chieh Lin

**Affiliations:** Department of Urology, MacKay Memorial Hospital, Taipei City, Taiwan (R.O.C.); Department of Pathology, Tamsui Branch, MacKay Memorial Hospital, New Taipei City, Taiwan (R.O.C.); School of Medicine, MacKay Medical College, New Taipei City, Taiwan (R.O.C.); Department of Urology, Hsinchu Branch, MacKay Memorial Hospital, No. 690, Sec. 2, Guangfu Rd., East Dist., Hsinchu City, Taiwan (R.O.C.)

**Keywords:** AAM, deep angiomyxoma, mesenchymal tumor, paratesticular mass, older

## Abstract

Aggressive angiomyxoma (AAM) is a rare mesenchymal myxoid tumor, and most cases occur in the pelvic region or perineum of adult females. AAM is very rare in males. Most of these cases have been diagnosed in patients aged 30–60 years, and the tumors involved the pelvic cavity, scrotum, or spermatic cord. AAM can mimic inguinal hernia, hydrocele, or paratesticular neoplasm. Four male cases have been reported with paratesticular AAM mimicking a testicular/epididymal tumor, and to the best of our knowledge, this is the oldest patient in the literature. Because of its rarity, making an exact diagnosis before surgery is difficult. Herein, we present a case of AAM in an 82-year-old man and review the literature.

## 1 Introduction

Aggressive angiomyxoma (AAM) is a rare mesenchymal tumor. In 1983, Steeper et al. [1] described nine cases of female pelvis and perineum tumors, which they called AAMs [2]. In men, less than 50 cases have been reported, involving the scrotum, pelvic region, perineum, and inguinal area [3]. AAM is difficult to diagnose without pathological proof, and misdiagnosis as hydrocele, spermatocele, testicular, and paratesticular neoplasia is common [2,4]. In the literature review, only four cases were identified. Herein, we report on an 82-year-old male patient who had paratesticular AAM, and to the best of our knowledge is the oldest case in the literature. Informed consent was obtained from the patient.

## 2 Case report

**Figure 1:**
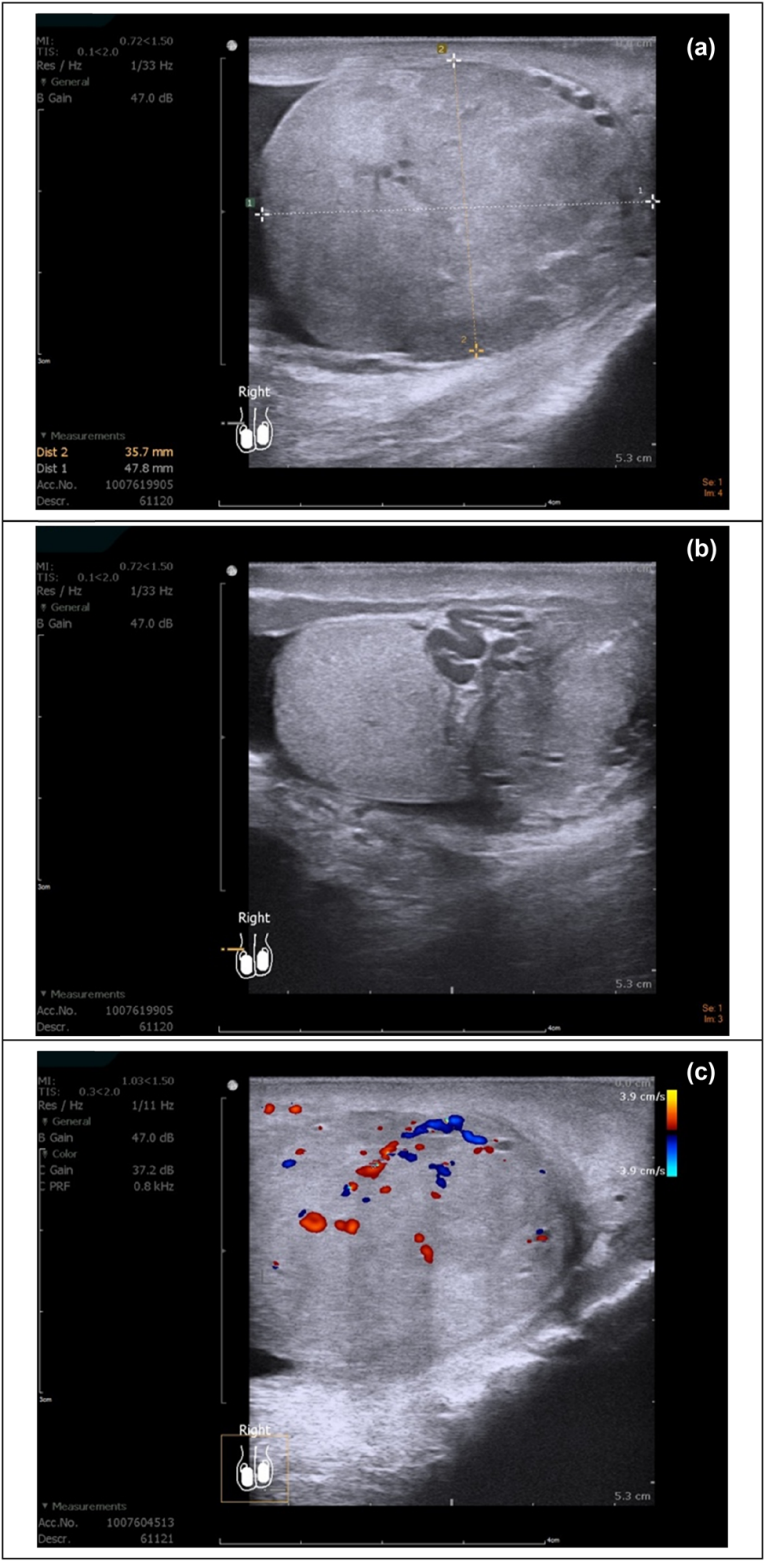
Ultrasound sonography: (a) a well-capsulated heteroechoic round mass over the tail of epididymis, 4.7 × 3.5 cm in size, and (b) abundant tortuous vessels adjacent to the paratesticular tumor. (c) The tumor had a rich blood supply on color Doppler sonography.

An 82-year-old man recently diagnosed with prostate cancer, which was under surveillance, presented to our outpatient department with a right palpable painless paratesticular mass. The physical examination revealed a round paratesticular mass that was firm in consistency without tenderness. It was fixed on the lower pole of the right testis and mimicked an epididymal or testicular neoplasm. Scrotal sonography showed a heterogenous round mass over the tail of epididymis (Figure 1a and b). Rich blood supply was noted under Color Doppler sonography (Figure 1c). The patient denied a history of cryptorchidism or previous trauma. Further imaging, including whole abdominal computer tomography scan and bone scan, showed no lymphadenopathy or distant tumor metastasis. The tumor markers Lactate dehydrogenase, beta-Human Chorionic Gonadotropin, alpha-fetal protein, and CEA were at normal levels.

**Figure 2:**
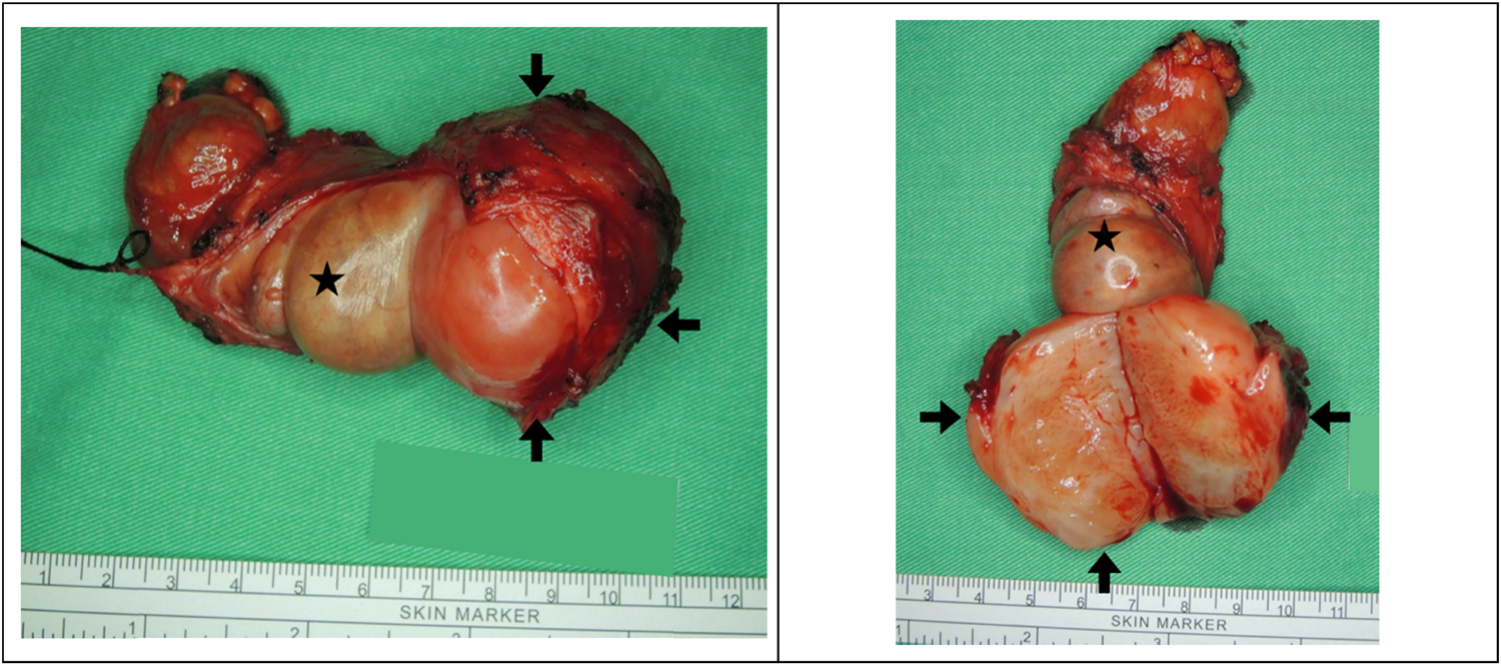
A white, grayish, well-circumscribed, solid tumor (4 × 3.5 cm) adherent to atrophic testis and epididymis (arrow: tumor; star: testis).

**Figure 3:**
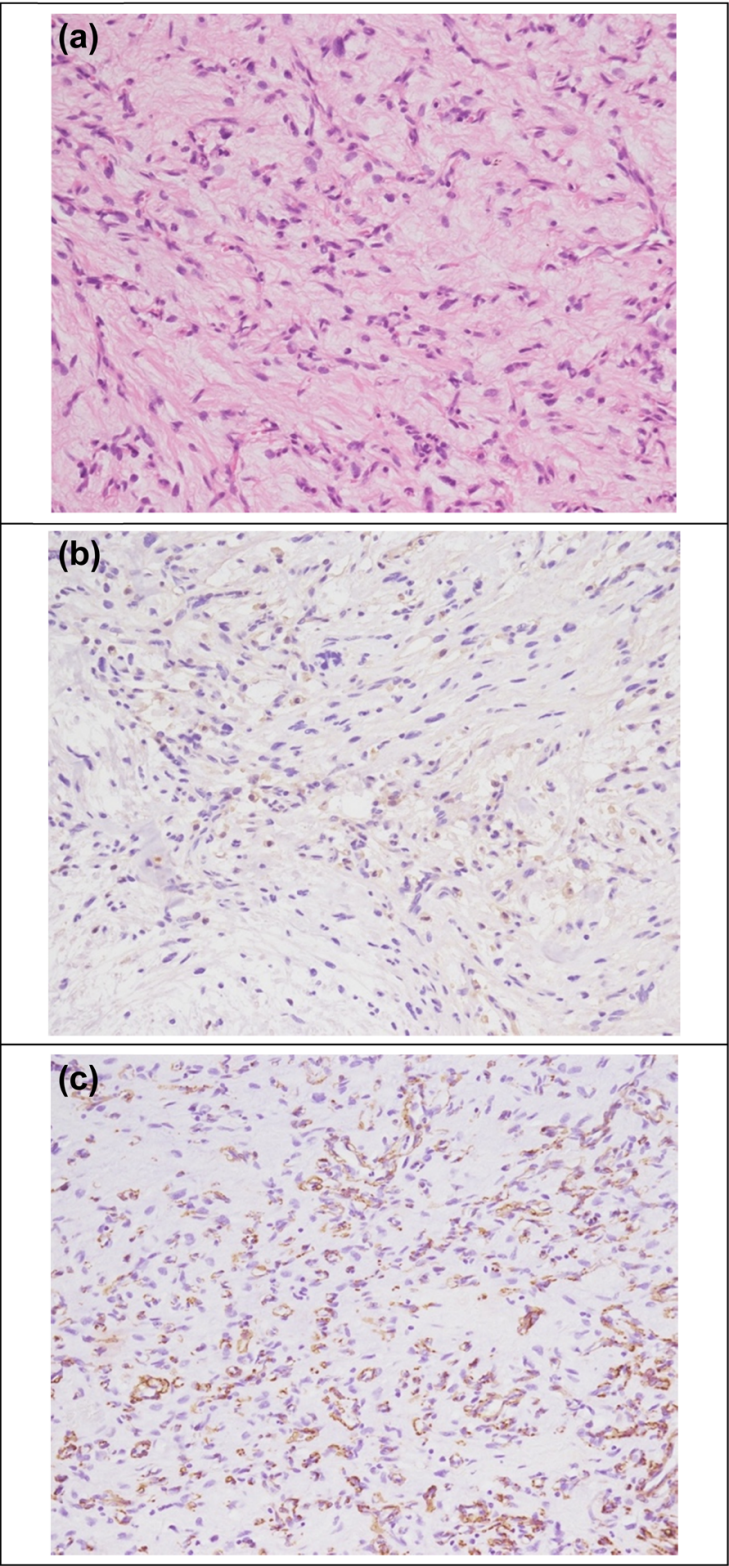
Typical bland spindle or stellate cells with little or no nuclear polymorphism and variably elongated cytoplasm set in a mucomyxoid stroma. Vascularity was variably composed of delicate to more hyalinized vessels (a) (hematoxylin and eosin, magnification 200×); typical cytoplasmic desmin (b) and smooth muscle actin (c) immunopositivity (magnification 200×).


Epididymal tumor excision by a scrotal approach was arranged. Because it presented as a non-testicular tumor, orchiectomy from the inguinal approach did not take into consideration before surgery. However, the operation was shifted to right orchiectomy because of severe tumor adhesion onto the testis. Gross examination showed a gray, round, and solid tumor with clear boundaries (Figure 2). Histologically, the testicular adnexal mass was a deep angiomyxoma composed of bland spindle cells in a myxoid matrix containing delicate vessels. The lesion was immunoreactive for CD34, smooth muscle actin, and desmin and negative for S100, estrogen receptors (ERs), and progesterone receptors (PRs) (Figure 3). The patient has been followed up for 4 months without disease recurrence. Long-term follow-up is needed for AAMs, we will arrange further images and physical examination at our out-patient department per 3 months initially. Our follow-up policy is scrotal echo every 6 months for 2 years and whole abdominal CT annually.

**Ethics approval and consent to participate:** Not applicable.
**Consent for publication:** Informed consent was obtained from the patient for publication of this case report and any accompanying images.

## 3 Discussion and conclusion

Most documented cases of AAMs have been reported in the genitals, perineal, and pelvic regions in women of childbearing age. In men, only case reports or case series have been reported. A male to female tumor ratio of 1:6 has been described in some studies [5]. The lesions usually originate from the scrotum, spermatic cord, perineum, or pelvic cavity [6]. A case of AAM in the right thigh has been reported [7]. The tumor is slow-growing [2]. The term “aggressive” was first described in female patients, and implies a characteristically locally infiltrative tumor with frequent recurrence [4]. Considering its benign nature, the nomenclature was changed from “aggressive” to “deep” in the Fourth Edition of the World Health Organization Classification of Soft Tissue tumors in 2013. The reason for frequent recurrence may be its location, which was difficult to resect with definite margins. Despite the frequent recurrence, distant metastasis has seldom been reported [2]. A literature review identified seven male cases in the scrotum and paratestis (Table 1)

**Table 1:** Clinical details of paratesticular angiomyxoma in a literature review

Source (author)	Site	Age	Size (cm)	Treatment	Immunostaining results	Follow-up (NED/LR)
Chen et al.*	Paratestis	82	4.7 × 3.5	Orchiectomy	CD34, Desmin, SMA(+) S100, ER, PR(−)	NED (4 months)
Neyaz et al. [2]	Paratestis	53	15 × 10	Orchiectomy	Vimentin, CD34, SMA(+) Desmin (focal+) S100, ER, PR(−)	NED (12 months)
Serao et al. [6]	Paratestis	72	7.7 × 5.6	Orchiectomy	CD34, Desmin, Actin, PR(+), ER(−)	NED
Aydin et al. [13]	Paratestis	66	11 × 7.5	Orchiectomy	CD34, ER, PR(+) SMA, S100 (−)	NED (6 months)
Ismail et al. [14]	Paratestis	65	11 × 8	Orchiectomy	Desmin, SMA(+) ER (focal+) PR, CD34, S100(−)	NED (24 months)
Durdov et al. [15]	Scrotum	37	7 × 5	Wide excision	Vimentin(+) Desmin(−) SMA(−) S100(−)	NED (24 months)
Chihara et al. [16]	Scrotum	47	17 × 10	Wide excision	ER(focal+) PR(−) S100(−) HHF35(−)	NED (14 months)
Kirkilessis et al. [17]	Scrotum	57	11	Wide excision	Desmin, CD34, SMA(+) ER(focal+) PR(focal+)	NED (24 months)

Abbreviations: NED, no evidence of recurrence; LR, local recurrence; SMA, smooth muscle alpha-actin; ER, estrogen receptor; PR, progesterone receptor (*our case).

According to literature reviews, the most common site of AAMs is the scrotum followed by the inguinal region. Most of the tumors were growing beside the spermatic cord [8]. For the paratesticular region, four other cases were presented. Only one patient is younger than 65 years. Because the tumor was usually asymptomatic, most tumors were more than 10 cm in size. All of them received orchiectomy for diagnosis and treatment. In the limited follow-up duration, no local recurrence was mentioned after excision with a clear margin.

As the tumor can occur at various sites, the diagnosis is difficult. It can mimic other non-neoplastic lesions including hydrocele, inguinal hernia, or testicular neoplasm [9]. Scrotal ultrasound is typically the primary imaging modality, and magnetic resonance imaging (MRI) can provide further details [3]. Sonography for AAMs demonstrates mixed echogenicity, lamellated appearance with alternating layers of hypo and hyperechoic tissues. Color Doppler demonstrates internal vascularity. On T2-weighted MRI, high signal intensity relative to the muscle with “swirled” low signal intensity bands within a hyperintense tumor has been noted [10]. However, typical ultrasound images to definitively describe AAMs are lacking. The imaging nowadays is not always clear in terms of benign tumors and this imposes a surgical excision as a treatment and diagnostic approach [11]. In our cases, MRI was not arranged before the operation, because AAMs had been diagnosed after excision. It will be an option if we find a similar tumor in the scrotum in the future.

Since none of these imaging modalities can reliably distinguish AAM from malignant tumors including sarcoma, the mainstay of treatment is the exploration and complete surgical resection [3]. The diagnosis and distinction of AAM from other benign and malignant myxoid soft tissue tumors are mainly based on morphology and immunohistochemistry. Immunoreactivity for desmin, SMA, vimentin, and CD34 is noted in tumor cells, at least focally [2]. A case report mentioned that the difference between males and females is the presence of ERs and PRs. In contrast to females, where ERs or PRs are almost always expressed, ER and PR stainings are usually negative in males. The authors suggested that these markers play a role in tumor development and pathogenesis, but do not apply to males [2]. In our case, ER and PR immunostaining were negative. It is possible that the expressions of ER and PR are related to tumor development and recurrence, and that frequent recurrences do not apply to males. ERs and PRs can help diagnose the AAMs in the immunohistological stain. However, the relationship of AAM with other hormones is not well described in the literature. The hormone profile surveying, such as pituitary, thyroid, parathyroid, adrenal, and sex hormone, will be considered, if we found similar AAMs in men with a positive result of ERs and PRs. The potential use of HMGA2 immunohistochemistry staining is also quite significant [12]. Although, we did not stain the HMGA2 for our case, it is a novel staining with quite sensitivity but not an entirely specific maker for deep angiomyxoma.

In men, AAMs should be considered in the differential diagnosis of paratesticular tumors. To the best of our knowledge, four paratesticular AAMs have been reported. It is a slowly growing benign tumor, but its locally aggressive and infiltrative nature makes it difficult to resect alone without orchiectomy. Although approximately 70% of cases locally recurred within the first 3 years [4], our review showed a lower rate of local recurrence of AAMs in males. Although AAMs in females occurred frequently in the pelvis area with easy local recurrence after excision, in men, no local recurrence was presented during short period follow-up. Wide excision with free margin is important for any tumor doubted malignancy. Due to its atypical presentation and rarity in males, we presented our clinical images and typical histologic findings for future references.

Although AAMs are rarely noted in men, however, if we find a well-circumscribed tumor in the scrotum, the diagnosis should be kept in mind. Furthermore, the infiltrated paratesticular tumor was slowly growing but it would be severe adhesive to the testis. So that, before wide excision, informed consent to the patient about the possibility of orchiectomy for clear margin is considered.
